# Does Urban Dialect Ability of Migrant Children Significantly Affect Academic Performance? Analysis of Mediating Effects Based on School Integration

**DOI:** 10.3390/bs15050645

**Published:** 2025-05-09

**Authors:** Yuelong Ming, Cixian Lv, Zihan Wang, Haoran Cui, Kejun Zhang, Xiaotong Zhi

**Affiliations:** School of Education Science, Qingdao University, Qingdao 266071, China; lvcixian@qdu.edu.cn (C.L.); wangzihan2@qdu.edu.cn (Z.W.); cuihaoran0527@163.com (H.C.); zhangkejun1@qdu.edu.cn (K.Z.)

**Keywords:** urban dialect ability, migrant children, school integration, academic performance

## Abstract

This study examined the relationship between the urban dialect ability and academic performance of migrant children, as well as the role of school integration between the two. This study collected data from 1687 migrant children aged 5–15 from 28 county-level units (counties, districts, cities) in China, sourced from the 2010 China Education Panel Survey (CEPS). The research results indicate the following: (1) the urban dialect ability of migrant children has a significant positive impact on their academic performance. (2) School identification, peer–peer engagement, and peer acceptance, as the three dimensions of school integration, play a mediating role between the urban dialect ability and academic performance of migrant children. Overall, this study provides theoretical support and policy recommendations for promoting school integration and academic performance development for migrant children.

## 1. Research Background and Problem Proposal

China is undergoing a process of rapid urbanization and large-scale population movements. In this context, the situation of educational equity for migrant children is of great concern. Migrant children are defined as children who migrate with their parents and live in new environments, facing challenges in accessing education and social services (Sime & Fox, 2015). The concept of equity, as defined by Levinson et al. (2022), encompasses five distinct aspects: equal outcomes for the overall population and each child, equal allocation of resources among students, schools, districts, states, or countries, equal experiences for each child, and individual growth levels. In recent years, the central government and the governments in the places of influx have formulated a series of policies aimed at guaranteeing the right to education of migrant children, and these relevant measures have made it possible to alleviate the problem of inequality in education among migrant children at the stage of school enrolment (Wu & Li, 2016). For example, some cities provide more equitable enrollment opportunities for migrant children through a points-based admission system (Y. Liu et al., 2021). However, studies have highlighted that upon arrival in the host city, the majority of migrant children live in highly segregated areas where public resources are scarce, due to family economic deprivation, language communication barriers, discrimination, etc. (Orfield & Lee, 2005). There is a huge gap in educational resources with local students, and opportunities to receive high-quality education are very limited (Potochnick & Mooney, 2015). Research has shown that the academic performance of migrant children is relatively worse than that of local students, which can directly or indirectly lead to parents and students frequently choosing to transfer schools or even drop out (Potochnick & Mooney, 2015). As the most important indicator for measuring the quality of basic education, students’ academic performance can not only predict their future labor market performance (Jeffrey & Daniel, 2002), Moreover, comparable academic performance in international assessments is closely related to the economic growth of various countries (Eric, 2013). Therefore, it is necessary to pay attention to the academic performance of migrant children.

China, as a vast and culturally diverse country, boasts its language, known as Mandarin, along with a variety of dialects that are commonly used in daily life. Urban dialect is a language form with local characteristics used by people in specific cities or regions in their daily lives. Consequently, while Standard Mandarin is designated as China’s official language, it is the local dialects that prevail in everyday interpersonal communication. A mastery of the local language is crucial for individuals to build relational networks and establish a cultural identity in unfamiliar living environments. This, in turn, influences their capacity to integrate into new settings (J. K. Liu & Zhang, 2020). Consequently, dialect serves as a vital social integration tool that can foster improved relationships between migrant children and their school interactions (Lu & Chen, 2019). For migrant children, schools in their new surroundings form the core of their activities, with teachers and classmates making up the bulk of their social interactions. The ability to navigate relationships within these groups directly dictates these children’s adaptation and behavior within the school environment (Roberts, 2006), culminating in superior academic performance (Pianta & Hamre, 2009). However, few studies have focused on the relationship between the ability of Chinese migrant children to speak local dialects in their destination cities and their academic performance, and the role of school integration between the two.

The purpose of this study is to analyze the effect of internal migration children’s urban dialect ability on their academic performance in the inflow city and the mediating effect of school integration between the two, using the China Education Panel Survey (CEPS) follow-up data.

## 2. Literature Review and Research Hypotheses

### 2.1. The Connotation and Influencing Factors of Academic Performance

Academic performance has rich connotations and extensions. Lamas’ (2015) study suggests that academic performance traditionally refers to some methods of expressing students’ learning levels, such as the level of a course, the average of a group of courses in a certain subject, or the average of all courses expressed in percentage or other quantitative ways. Donnelly et al. (2016) argue that academic performance is the degree to which students, teachers, or educational institutions achieve their educational goals. This study suggests that the academic performance of Chinese migrant children refers to their academic performance in the courses they study.

Previous research has explored the factors influencing students’ academic performance at four levels: individual students, family background, peer influences, and the school (classroom) environment (Dahl & Lochner, 2012; Lee & Shute, 2010). At the individual level of students, discussions mainly focus on cognitive and noncognitive factors (Privado et al., 2024). Cognitive factors have a strong correlation with academic performance. Meanwhile, studies have shown that after controlling for factors such as family background and individual cognitive factors, an individual’s noncognitive factors have a significant positive impact on their academic performance (Nagaoka, 2012). According to the school (class) environment theory, the teaching staff, educational conditions, and peer acceptance of a school can affect students’ academic performance (Jonah, 2004). In addition, research suggests that cognitive mapping and country of origin are important factors to consider when it comes to understanding the academic performance of immigrant students (Figueiredo, 2022).

### 2.2. Urban Dialect Ability and Academic Performance

This study suggests that the urban dialect ability of migrant children refers to their proficiency in using the local language of their migration. There is ample empirical evidence supporting the fact that children who are proficient in dialect skills perform well in various fields. A study conducted by Washington (2001) discovered that differences in dialect played a significant role in the consistent underperformance of African American students, compared to their white peers, in reading, math, and science. Simultaneously, research has consistently demonstrated that students who possess fluency in a standard dialect achieve significantly higher scores in reading compared to their counterparts who do not possess such fluency (Reaser & Adger, 2008). It is widely acknowledged by educators and sociolinguists alike that appreciating and valuing dialects within the classroom setting can exert a significant influence on the academic accomplishments of students (Cheshire, 2007). Additionally, B. Bernstein’s code theory proposes a sociological perspective on the relationship between children’s language use behavior and academic performance; that is, children who use different language codes have different school adaptation abilities, which directly or indirectly affect their academic performance (Collins & Bernstein, 1978). Dialects, being a subset of the lingua franca, serve as a means of strengthening one’s identity and facilitating interactions among members of society (Kalra & Danis, 2024). Migrant children’s proficiency in the local language in the city they move to can be used to construct not only the speaker’s self-identity (Syuhudi, 2019) but also helps them communicate smoothly with their school teachers and peers, which in turn effectively contributes to building strong interpersonal relationships (Jeong, 2011).

In summary, some scholars have studied and demonstrated that dialect proficiency has a positive impact on academic performance, while others have studied and demonstrated that dialect proficiency has an indirect positive impact on academic performance. Therefore, this study proposes Hypothesis 1: The urban dialect ability of migrant children has a significant positive effect on their academic performance.

### 2.3. School Integration and Its Mediating Role

#### 2.3.1. School Integration

Integration is defined as the process of individuals or groups infiltrating and merging with each other, ultimately integrating into mainstream society through contact, competition, adaptation, and other means (Park & Burgess, 1970). The theory of pluralistic integration suggests that integration should consider the diversity of the directions, processes, and outcomes of migrants’ integration in the cities where they flow in (Singh et al., 2014). Therefore, this study believes that school integration refers to the process of two-way interaction and mutual acceptance between migrant children and the destination school. Based on the previous analysis, scholars generally believe that school integration can be divided into the following dimensions: (1) subjective integration, including collective identity and school satisfaction; (2) objective integration, covering teacher–student relationships, peer relationships, academic performance, behavioral norms, and interpersonal harmony (Cava et al., 2015). Therefore, this study operationalizes school integration into three dimensions: school identification, peer engagement, and peer acceptance.

#### 2.3.2. The Mediating Role of School Integration

The language competence of migrant children in the inflow city is an effective means of achieving social integration. On the one hand, familiarity with the local language facilitates migrants’ faster access to support and assistance from the local group when they first arrive in the influx (Kearns & Whitley, 2015). On the other hand, the ability to have similar language habits facilitates the children of immigrants to develop identity and school identification with the local group (Cotter & Horesh, 2015). According to language identity theory, groups within the same linguistic community have greater similarities in cognitive, attitudinal, and affective aspects as well as external exclusion. Linguistic integration is a key technique for migrant children to win peer acceptance in urban schools in their place of inflow (Gee & Ponce, 2010). Some scholars have discovered that individuals who speak the same language form communicative networks among themselves, with close relationships and high levels of identity among members (Kalra & Danis, 2024). It can be seen that migrant children with a high level of local dialect are not only able to establish a complete and good network of interpersonal relationships but also facilitate their integration at school.

In 1966, Coleman’s research significantly found that students’ academic performance improved due to racial integration (Coleman et al., 1966). School integration, devoid of class and racial segregation, enables underprivileged students to benefit from the economic, social, and cultural “capital” of middle-class families (Blum, 2019). This has led to widespread concern about school integration. Subsequent to Coleman’s report, numerous studies, drawing from social interaction theory, have examined the mechanism through which school integration impacts students’ academic performance, focusing on the theory of “peer group effects” (Bain & Anderson, 1974). Bacete et al. (2021) determined that peer relationships and academic performance exhibit stability during childhood, while Warrican et al. (2019) discovered substantial peer effects in mathematics, reading, and science, even after accounting for individual demographic variations. Moreover, Henderson et al. (1978) found that positive peer relationships significantly contribute to children’s cognitive development and academic achievement, potentially due to the supportive nature of peer engagements, which enhance task-oriented learning (Kiuru et al., 2014). Empirical studies have also revealed that high-quality peer engagement partially mediates the positive influence of the home environment on the academic achievement process (Zhao & Zhao, 2022). In addition to peer recognition, extensive research has also shown that school identification can have an impact on academic performance. School identification and school belonging are positively related to students’ final academic performance (Korpershoek et al., 2019), and students’ psychological identification with school mediates the relationship between perceptions of school climate and writing and arithmetic performance (Maxwell et al., 2017). In summary, school integration has an impact on students’ academic performance. Therefore, this study proposes Hypothesis 2: School integration of migrant children mediates the relationship between urban dialect ability and academic performance.

**Figure 1:**
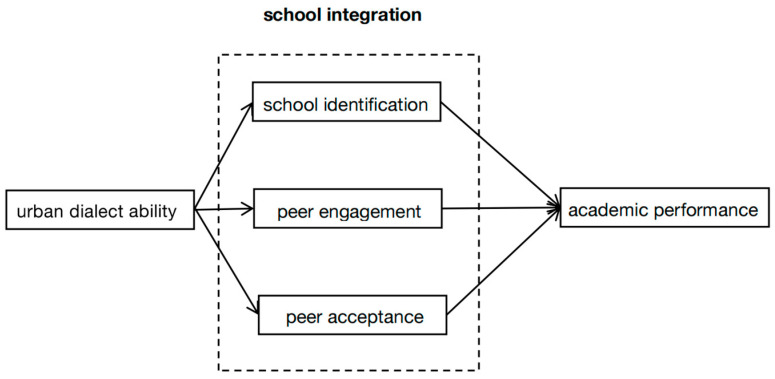
Conceptual dimensions.

In summary, this study formulated the above hypotheses and constructed a mediation model diagram with a conceptual dimensionality map (see Figure 1).

## 3. Materials and Methods

### 3.1. Participants

This paper uses data from the 2010 baseline data questionnaire for children aged 5 to 15 years from the China Education Panel Survey (CEPS) designed and completed by the National Survey Research Center at Renmin University of China. This survey randomly selected 28 county-level units (counties, districts, cities) from China as survey points. A total of 112 schools and 438 classes were randomly selected from the selected county-level units for investigation. All students in the selected classes were sampled, and a total of about 20,000 students were surveyed.

Figueiredo et al. (2019) studied the learning styles and test results of different immigrant students. In this study, “immigrant” refers to nonlocal students studying Portuguese as a second language in Portuguese schools, who may come from different national backgrounds. For the subject of this study, ‘migrant children’, are defined as school-age children and adolescents whose registered residence is in rural areas of other provinces (districts and cities) and counties (districts) of the province, and whose migrant parents go to the urban areas, towns, and districts (cohabitation) of the destination and receive compulsory education in school. Therefore, based on the requirements of the target population, the appropriate study population was selected through the following questions, “Where are you registered (i.e., do you live in this county)”, and “Do you live with anyone in your current home (i.e., do you live with your parents)”. These screenings identified 1687 migrant children. In the survey sample, 47.4% of the mobile children were male and 52.6% were female. Overall, 32.0% of mobile children were only children and 68.0% were not.

### 3.2. Tools

#### 3.2.1. Urban Dialect Ability

Regarding the measurement of dialect ability, most scholars use testing methods such as pronunciation, listening, comprehension, etc. (Díaz-Campos, 2004). The measurement of urban dialect ability by CEPS only includes one question: “Do you know the dialect of the city you are moving to?” The response options were “don’t understand it at all”, “understand it, but don’t speak it”, “speak it only a little”, and “speak it basically, but at an average level”. The five levels of “can speak, very fluent” cover the whole process, from listening and speaking to understanding. The recorded score is 1–5 points. Given the experience of previous scholars using this question item to measure dialect ability (He & Cai, 2023; Ding, 2022), this study uses this question item and considers the first three items as weak dialect ability, and the last two items as strong dialect ability, encoded as 0 and 1, respectively.

#### 3.2.2. Academic Performance

Academic performance, as a measurable variable, can be defined as the measurable outcomes demonstrated by students in their academic performance, with a focus on academic achievement (Marilou & Thelma, 2023). This study selected three measurement indicators based on the above literature, and the question is as follows: What were the Chinese/mathematics/English scores in the 2014 autumn midterm exam? In this study, Cronbach’s α for the academic performance was 0.878.

#### 3.2.3. School Integration

In this study, school integration was operationalized into three dimensions: school identification, peer engagement, and peer acceptance. Among them, school identification is measured by the mobile children’s emotional belonging status to the school. Peer engagement was measured by the status of the migrant children’s network of urban children’s peers in the school. Peer acceptance is measured by the acceptance and recognition of migrant children by urban children in the inflow area. The School Integration Scale for Migrant Children has 11 measures. For the school identification dimension, the questions are as follows: I wish I could go to another school (reverse scoring), I regularly participate in school or classroom-organized activities, and I feel close to the people in this school. For the peer engagement dimension, the questions are as follows: How many good friends do you have in school with you? For the peer acceptance dimension, the questions are as follows: Are the students in your class from this county (district) willing to become friends with the students from cities in other counties (districts)? Are the students in your class from this county (district) willing to be friends with the students from rural areas in other counties (districts)? Most of the students in my class are friendly to me. Cronbach’s α in this study was 0.654 for the School Integration School Identification Scale, 0.779 for the School Integration Peer Engagement scale, 0.779 for the School Integration Peer Acceptance Scale, and the CITC values for each item were above 0.5. The validated factor model of school integration was constructed, and the reliabilities of its three dimensions were 0.761, 0.615, and 0.790, respectively, the construct validities were 0.644, 0.641, and 0.764, respectively, and the first-order three-factor validated factor model generally fit the formal sample data.

#### 3.2.4. Data Analysis

This study used SPSS 26 to first confirm the association between urban dialect ability, school integration, and academic performance among migrant children by correlation analysis before evaluating the proposed model. Since the independent variable of urban dialect ability is a binary categorical variable, linear regression is used to test the mediation model using the “stepwise method” (Mackinnon et al., 1995; Wen & Ye, 2014), the focus was on studying the mediating role of school integration between urban dialect proficiency and academic performance. Since students, teachers, and schools have a major impact on coursework performance and acceptance, this study split the control variables into student characteristic factors, teacher characteristic variables, and school characteristic variables for the regression analysis. The student characteristic variables include demographic variables such as gender and whether or not the student is an only child. The teacher characteristic variables contain the age, gender, education, and title of teachers. The school characteristics variables, including whether the school was in the central area of the city, school quality ranking, and the percentage of local students in the city, were included in the regression model as control variables.

## 4. Results

### 4.1. Correlation Analysis

Since urban dialect ability is a categorical variable, and school identification, peer engagement, peer acceptance, and academic performance are all continuous variables, the Point Qualitative Correlation Coefficient is used for correlation analysis.

First, there was a positive correlation between urban dialect ability and academic performance (r = 0.177, *p* < 0.001). Second, there was a statistically significant positive correlation between academic performance and school identification, peer engagement, and peer acceptance in school integration (r = 0.020, *p* < 0.01; r = 0.032, *p* < 0.001; r = 0.027, *p* < 0.001). Finally, there were significant positive correlations between urban dialect ability and school identification, peer engagement, and peer acceptance in school integration (r = 0.087, *p* < 0.01; r = 0.010, *p* < 0.01; r = 0.037, *p* < 0.01). The specific information is shown in Table 1.

### 4.2. Regression Analysis

**Figure 2:**
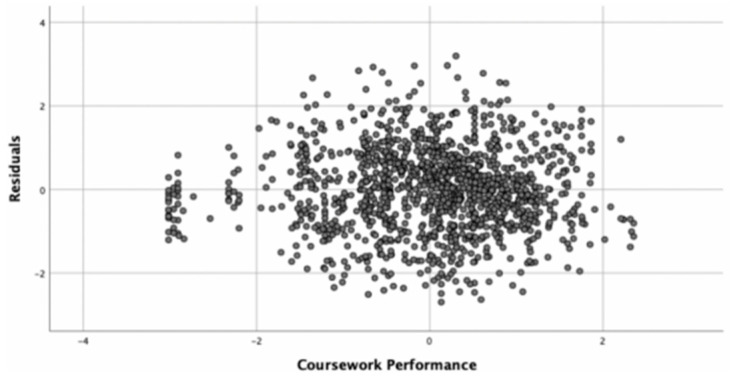
Scatter plot of partial residuals.

This study requires solving the issues of multi-collinearity, serial correlation, and heteroscedasticity of the model for the multiple linear regression model to produce scientifically solid explanations. The multicollinearity test is performed by tolerance and the variance inflation factor (VIF). The VIF of each variable in each model of this study ranged from 0 to 3, indicating that there was no multicollinearity problem. Since this study does not involve the comparison of sample values over multiple periods, and the Durbin–Watson (DW) values of each regression model are close to 2, there is no serial correlation problem in each model. The scatter plot of the residual term is used to determine the heteroskedasticity problem. Because the scatter plot of the residual term of each regression model in this study reveals a disordered state, and the heteroskedasticity problem is mostly found in time series data, this study concludes that there is no heteroskedasticity problem in this study (see Figure 2).

#### 4.2.1. Regression Analysis of Urban Dialect Ability on Academic Performance and School Integration of Migrant Children

Academic performance, school identification, peer engagement, and peer acceptance were used as outcome variables, respectively, and control variables (student characteristics variables, teacher characteristics variables, school characteristics variables) and independent variables were added sequentially. The urban dialect ability of migrant children was regressed and analyzed. Table 2 shows the regression results, research revealed that the urban dialect ability of mobile children had a significant positive effect on academic performance (*β* = 1.502, *p* < 0.001). The overall effect is significant, and Hypothesis 1 holds.

In addition, school identification has a significant positive impact on academic performance (*β* = 0.574, *p* < 0.001), peer engagement has a significant positive effect on academic performance (*β* = 0.797, *p* < 0.01), and peer acceptance significantly and positively affects academic performance (*β* = 0.638, *p* < 0.001). Therefore, school integration has a significant positive impact on academic performance.

#### 4.2.2. Analysis of the Mediating Effect of School Integration on Urban Dialect Ability and Academic Performance

Table 3 reflects the structure of the test for mediating the effects of school integration on urban dialect ability and coursework performance. This table uses the academic performance of migrant children as the dependent variable. Model 1 shows that after adding the school identification variable, urban dialect ability still has a significant positive impact on academic performance, and school identification significantly positively affects academic performance (*β* = 0.560, *p* < 0.001; *β* = 1.639, *p* < 0.001). Therefore, school identification plays a mediating role between urban dialect ability and academic performance. Model 2 shows that after adding the peer engagement variable, urban dialect ability still has a significant positive impact on academic performance, and peer engagement significantly positively affects academic performance (*β* = 0.848, *p* < 0.001; *β* = 0.821, *p* < 0.01). Therefore, peer engagement plays a mediating role between urban dialect ability and academic performance. Model 3 shows that after adding the peer acceptance variable, urban dialect ability still has a significant positive impact on academic performance, and peer acceptance significantly positively affects academic performance (*β* = 0.983, *p* < 0.001; *β* = 0.875, *p* < 0.01). Therefore, peer acceptance plays a mediating role between urban dialect ability and academic performance. In summary, school integration plays a mediating role between urban dialect ability and academic performance, and hypothesis 2 holds.

## 5. Discussion

International researchers have predominantly focused on the impact of language skills in the host countries of international migrants on their labor market performance, encompassing employment rates and wages (Chiswick & Miller, 1995; Dustmann & Fabbri, 2003; Yao & Van Ours, 2015; Zhu & Grigoriadis, 2022). A limited number of researchers have started to recognize the correlation between language skills and educational aspects among migrant children (Christian et al., 2010; Geay et al., 2013; Yao et al., 2016; Jain, 2017). This study focuses on the relationship between urban dialect ability and the education of migrant children and finds that urban language ability has a significant positive impact on the academic performance of migrant children, and Hypothesis 1 holds, which is consistent with previous research results. The use of regional dialects rather than standard languages has also been demonstrated to improve learning outcomes in a study of Austrian junior high school students (Rey & Steib, 2013). Dialect transfer can be an efficient method for improving the academic performance of students who speak non-dominant dialects, such as African American English (AAE) (Byrd & Brown, 2021).

Exploring the influencing mechanism, we found that school integration plays a mediating role between the two, and Hypothesis 2 holds. Previous studies have demonstrated that Shenzhen migrant children who speak local dialects are more likely to identify as urbanites than migrant children who can only understand local dialects (H. S. Liu & Jin, 2018). For Shanghai natives, proficiency in Shanghainese implies civilization, familiarity with city life, and membership within the Shanghainese community (Xu & Liu, 2022). Internationally, studies have found immigrant populations in rural areas of Finland where Swedish is the official language. The study explores how these immigrants construct their own identities and the process of acquiring local dialects. During the process of adapting to a new environment, one will shape their local identity through language changes (Ekberg & Stman, 2020). This may be because individuals’ potential instrumental and expressive functions are often hidden in language-based interactions (Ruan, 2017). A mastery of the local language is crucial for individuals to build relational networks and establish a cultural identity in unfamiliar living environments.

According to Bernstein (1959), the founder of symbolic theory, language plays a role in distinguishing social class as it encompasses intrinsic distinctions about identity latent in its constitution and usage. This study once again confirms this viewpoint. When migrant children attain proficiency in the language of their migration destination, they acculturate into a group of peers who possess a standardized linguistic expression system. This convergence of interests and values leads to the development of an “in-group” identity, augmenting the likelihood of receiving social support from classmates (Platow et al., 2012). Consequently, the local language often becomes a means of identification in peer interactions, and mobile children with limited language adaptation skills become susceptible to being labeled as “out-group” members, consequently experiencing interactive exclusion. Throughout this process, the community members’ displays of hostility, indifference, or genuine acceptance play pivotal roles in the school integration process and outcomes for migrant children. Students who achieve positive school integration not only exhibit a strong sense of identification with their educational institution but also experience an increase in their sense of belonging to a collective, subsequently improving their learning style and academic performance (Smyth et al., 2017).

### 5.1. Theoretical Implications

One of the contributions of this study is a novel perspective on the impact of urban dialect ability among migrant children as the main independent variable on academic performance. The second contribution of this study is to investigate the influence of urban dialect ability on the academic performance of migrant children from the standpoint of school integration. It aims to explore the mediation mechanism between school integration and academic performance and improve the understanding of the mediating chain of “urban dialect ability–school integration–academic performance”. The final contribution of this study emphasizes the practical significance of achieving educational equity and social mobility for migrant children. At the micro level, the academic performance of migrant children is closely related to their future higher education attainment, human capital accumulation, and job market performance. Meanwhile, at the macro level, the academic performance and educational attainment of migrant children, as a vulnerable group, have the potential to challenge socioeconomic stratification and facilitate social mobility.

### 5.2. Practical Implications

Based on these findings, we put forward some practical suggestions:

Firstly, by adopting a linguistic sociological perspective, we should comprehensively investigate the non-market benefits of language adaptability. We should pay attention to how this adaptability can promote the academic performance and classroom integration of immigrant children globally. This not only involves traditional immigration-receiving countries such as Europe and North America but also covers emerging immigration destinations such as Asia and Africa. On one hand, schools should strengthen the supervision of teachers who provide standard native language instruction while also encouraging students to communicate more frequently in the standard native language. Previous studies have shown that mobile-assisted language learning has a positive impact on language learning outcomes, particularly in vocabulary acquisition, grammar knowledge, and listening comprehension (Figueiredo, 2023). Therefore, mobile-assisted language learning can be used to enhance students’ urban dialect ability. On the other hand, special activities and other means should be utilized to enhance the students’ recognition of school and assist mobile children who encounter difficulties in school integration due to dialect resistance. Simultaneously, teachers play a direct role in facilitating the school integration process for migrant children. In a broader educational environment, such as working with immigrants or immigrant children from different Spanish- or Arabic-speaking countries around the world, the role of teachers is particularly important. In their daily educational practices, teachers should prioritize addressing any learning style or peer engagement issues arising from language differences. They should employ various teaching techniques and organize corresponding class activities to ameliorate the learning challenges faced by migrant children and enhance emotional communication between migrant children and local students. Lastly, a precise language support system should be implemented, catering to the specific needs of each district, in order to strike a balance between the usage of language codes, namely the standard native language and urban dialects. Language support activities should be tailored according to the dominant languages spoken in different regions, and projects promoting local culture should also be implemented.

### 5.3. Limitations and Future Directions

This study utilized the China Education Panel Survey (CEPS) to examine the academic performance of migrant children from a linguistic perspective. However, certain issues arose from the omission of variables due to the limited nature of the survey’s question items. Firstly, this study failed to consider the external impacts of varying language environments on migrant children. For instance, it overlooked the heterogeneity between northern and southern accent environments and their influence on the language adaptability of migrant children. It is essential to account for external factors, including geographic considerations. Secondly, this study did not take into account the effects of dialect identity among migrant children. It is crucial to recognize how children’s subjective perceptions of dialects can shape their attitudes toward receptive learning (Ryneland & Jensen, 2020). Thirdly, although this study controlled for migration distance, it lacked information regarding the specific places of migration. Consequently, it failed to consider the heterogeneity in language adaptability resulting from distinct contexts, such as urban areas dominated by the standard native language versus rural areas dominated by dialects. Despite the aforementioned limitations of the CEPS, this study managed to incorporate a more comprehensive range of control variables, which substantially alleviated the endogeneity problem. Future research should focus on diverse language domains and explore additional pathways to pinpoint precise and in-depth causal mechanisms influencing the academic performance of migrant children within specific dialect areas.

## 6. Conclusions

This research investigated the influence and impact of dialectal competence on academic performance within the framework of the school integration challenges faced by migrant children. The findings revealed the following: (1) The urban dialect ability of migrant children has a significant positive effect on their academic performance. This discovery expands the traditional educational equity theoretical framework from a new dimension of language capital. Research has shown that after controlling for variables such as students, teachers, and schools, dialect proficiency can still independently explain variations in academic performance, providing a new explanatory path for understanding the educational disadvantages of marginalized student groups. (2) The school integration of migrant children mediates the relationship between urban dialect ability and academic performance. Specifically, dialect proficiency promotes academic success through a triple socialization mechanism: firstly, increasing school identification and sense of belonging; next, promoting close peer communication and establishing good interpersonal relationships; finally, enhancing peer acceptance and forming internal group identity.

## Figures and Tables

**Table 1:** Correlation analysis of variables.

Variables	Urban Dialect Ability	School Identification	Peer Engagement	Peer Acceptance	Academic Performance
**Urban Dialect Ability**	1				
**School Identification**	0.087 ***	1			
**Peer Engagement**	0.010 **	0.164 ***	1		
**Peer Acceptance**	0.037 **	0.363 ***	0.164 ***	1	
**Academic Performance**	0.177 ***	0.020 **	0.032 ***	0.027 ***	1

*Note*. *N* = 1687; ** *p* < 0.01 and *** *p* <0.001.

**Table 2:** Regression analysis of urban dialect ability on academic performance and school integration of migrant children.

Variables	Academic Performance		School Identification		Peer Engagement		Peer Acceptance	
	*β*	*t*	*β*	*t*	*β*	*t*	*β*	*t*
**Student Gender**	−0.196	−2.013	0.062	1.425	0.031	0.313	0.009	0.437
**Only Child or Not**	−0.158	−1.621	−0.070	−1.571	0.004	0.036	−0.028	−1.353
**Whether or Not to Live on Campus**	0.235	2.414	0.105	1.877	0.227	2.043	−0.015	−0.596
**School Ranking**	0.355	3.646	0.001	0.012	0.076	0.684	0.001	0.035
**School Location**	−0.254	−2.606	0.061	1.976	−0.224	−2.016	0.015	1.039
**Percentage of Urban Students**	−0.763	−7.836	−0.120	−3.277	0.117	1.053	−0.014	−0.809
**Teacher Education**	0.831	8.534	−0.001	−0.022	0.005	0.045	0.003	0.346
**Teacher Title**	1.342	13.786	0.021	0.471	0.014	0.126	0.033	2.011
**Faculty Gender**	0.128	1.313	0.134	3.007	0.021	0.189	−0.042	3.487
**Teacher Age**	−0.164	−1.683	−0.021	−0.471	0.016	0.144	0.434	21.111
**Urban Dialect Ability**	1.502	15.424 ***	0.574	12.882 ***	0.797	7.173 **	0.638	31.66 ***
**Δ*R^2^***	0.637		0.714		0.682		0.745	
***F***	145.692 **		256.941 ***		197.453 ***		345.620 ***	

*Note.* ** *p* < 0.01 and *** *p* < 0.001.

**Table 3:** Mediating effect test.

Variables	Academic Performance					
	Model 1		Model 2		Model 3	
	*β*	*t*	*β*	*t*	*β*	*t*
**Student Gender**	0.015	0.013	0.210	0.158	0.163	0.136
**Only Child or Not**	0.722	0.663	0.355	0.264	0.829	0.762
**Whether or Not to Live on Campus**	1.360	0.972	1.249	0.743	1.161	0.832
**School Ranking**	−0.068	−0.089	0.470	0.501	−0.127	−0.167
**School Location**	−2.722	−7.031 ***	−2.528	−5.326 ***	−2.734	−7.067 ***
**Percentage of Urban Students**	0.020	0.702	−0.014	−0.402	0.020	0.715
**Teacher Education**	5.655	6.158	5.003	4.582 ***	5.905	6.520 ***
**Teacher Title**	1.183	0.147	1.284	0.963	1.653	1.240
**Faculty Gender**	0.165	0.099	0.143	0.107	0.212	0.170
**Teacher Age**	0.241	0.121	0.263	0.197	0.142	0.114
**Urban Dialect Ability**	0.560	0.423 ***	0.848	0.636 ***	0.983	0.787 ***
**School Identification**	1.639	1.226 ***				
**Peer Engagement**			0.821	0.616 **		
**Peer Acceptance**					0.875	0.700 ***
**Δ*R^2^***	0.742		0.792		0.640	
***F***	329.807 ***		512.934 ***		147.376 ***	

*Note.* ** *p* < 0.01 and *** *p* < 0.001.

## Data Availability

The data presented in this study are available on request from the corresponding authors.
